# Emergence of Incidentalomas Following Chest CT Screening for COVID-19 Infection: A Multicenter Cross-Sectional Study

**DOI:** 10.7759/cureus.71861

**Published:** 2024-10-19

**Authors:** Nasser A Abunamous, Akram Takelah, Mohamed Abdilsalhen, Amel Alameeri, Shamma Al Nokhatha

**Affiliations:** 1 Internal Medicine, Tawam Hospital, Al Ain, ARE; 2 Pulmonary, Tawam Hospital, Al Ain, ARE; 3 Rheumatology, Tawam Hospital - College of Medicine and Health Sciences, UAE University, Al Ain, ARE

**Keywords:** covid-19, covid-19 infection, ct chest, interstitial lung disease, lung incidentalomas, lung lesion

## Abstract

Background

The COVID-19 pandemic has raised several questions about its potential long-term impacts. During the pandemic, computed tomography (CT) chest scans were frequently employed for the diagnosis of COVID-19 pneumonia. The increased utilization of CT scans as a diagnostic tool has facilitated the detection of subtle abnormalities that may not have been easily discernible previously.

Aim

The primary objective of the present study was to investigate the prevalence of non-COVID-19 lung incidental pathologies in chest CT scans performed to screen for COVID-19, with a focus on autoimmune conditions related to interstitial lung disease (ILD) following COVID-19 infection, as determined by positive chest CT results.

Methods

This retrospective observational study included all adult patients (aged ≥ 16 years) in Al Ain, a city in the United Arab Emirates, between June 2020 and June 2021. Patients who underwent high-resolution computed tomography (HRCT) or chest CT during this timeframe and exhibited lung pathologies beyond the typical changes associated with COVID-19 infection followed by at least one pulmonary consultation were eligible for inclusion while all typical COVID-19-related changes reported in lung pathologies were excluded from consideration in this study. The hospital's electronic medical system was used to obtain patient information and subsequent management approaches.

Results

Among a total of 3,000 CT scan reports, 318 individuals fit our inclusion criteria. Their mean age was 63 years, and 52% were female (n = 165). Of the patients, 12% (n = 38) were smokers and 17% (n = 54) were ex-smokers. A total of 231 (72.6%) of the patients exhibited incidental lung nodules while 87 (27.4%) displayed lung pathologies other than lung nodules, with 75 (23.6%) being diagnosed with pleural effusion, 63 (19.8%) with bronchiectasis, and 19 (5.9%) with emphysema. Furthermore, three patients (0.9%) had cavitary lung lesions and one was diagnosed with tuberculosis while two others were undergoing surveillance follow-up. Only one patient (0.3%) was identified with a lung mass, which was attributed to primary lung adenocarcinoma. The remaining eight patients (2.5%) had ILD findings (two had non-specific interstitial pneumonia, five had usual interstitial pneumonia, and one had hypersensitivity pneumonitis). All of the patients with ILD findings underwent investigations for autoimmune-related ILD; however, no cases of autoimmune-related conditions were identified during the subsequent follow-up.

Conclusions

This cross-sectional chest CT-based study provides insights into incidental lung abnormalities. A small percentage (10.6%) of the participants exhibited lung incidentalomas.

## Introduction

Incidental findings, or "incidentalomas," have become a common occurrence in CT scans, often revealing unexpected abnormalities. The rise in the use of advanced imaging techniques, especially during the COVID-19 pandemic, has made such findings even more frequent, playing a pivotal role in guiding subsequent medical decisions. High-resolution computed tomography (HRCT) chest scans were widely used to diagnose COVID-19 pneumonia, and their increased usage has uncovered subtle abnormalities that might have previously gone unnoticed [1]. A key debate remains: Should these incidental findings be addressed immediately or simply monitored over time?

This study aims to explore the prevalence of non-COVID-19-related lung pathologies detected in CT chest scans performed for COVID-19 screening. In particular, it focuses on identifying autoimmune conditions, such as interstitial lung disease (ILD), that may emerge following COVID-19 infection, as revealed by positive CT results.

## Materials and methods

This retrospective observational study covered all adult patients (aged 16 years and older) in Al Ain, United Arab Emirates, from June 2020 to June 2021. Ethical approval was granted by the Abu Dhabi Health Research and Technology Ethics Committee.

Inclusion criteria

The study focused on cases with incidental findings beyond the typical COVID-19-related lung changes, specifically examining autoimmune conditions associated with ILD following COVID-19. These cases were identified through positive chest CT scans and at least one pulmonary consultation.

Exclusion criteria

Cases exhibiting only typical COVID-19-related changes in lung pathology were excluded from this study.

Data collection involved reviewing medical records to identify individuals with documented positive COVID-19 polymerase chain reaction tests. Relevant information, including diagnostic details, imaging results, laboratory findings, and management, was systematically compiled. A total of 318 patients were included in the study. Descriptive analyses were used to summarize the data, providing insights into the clinical characteristics and outcomes of the patients.

## Results

The demographics of the participants are presented in Table 1.

**Table 1:** Demographics of the participants.

Baseline characteristics (n = 318)	Number (N)	Percent (%)
Age (range: 18-100) SD: 18.5		
Age groups		
18-40	61	19
41-60	132	42
61-100	125	39
Gender		
Male	152	48
Female	166	52
Smoking status		
Active smoker	40	12
Ex-smoker	54	17
Non-smoker	215	68
Unknown	9	3

Comorbidities and pre-COVID symptoms

In this study, the most common comorbidities were cardiovascular disease, affecting 30% (n = 127) of participants, followed by diabetes in 28% (n = 121) and hypertension in 25% (n = 105). Chronic lung diseases, such as asthma or chronic obstructive pulmonary disease, were observed in 17% (n = 71) of the participants. Prior to their COVID-19 diagnosis, 49.7% (n = 158) experienced shortness of breath, while 18.9% (n = 60) had a cough, and 0.6% (n = 2) reported weight loss. The remaining 50.3% (n = 160) of cases were asymptomatic.

Main CT findings

The most frequent incidental finding in the study was lung nodules, seen in 56% of patients. Other lung pathologies were observed in 87 patients (27.4%), including pleural effusion in 75 patients (23.6%), bronchiectasis in 63 (15%), and emphysema in 19 (5%). Additionally, three patients (0.9%) had cavitary lung lesions, one patient (0.3%) was diagnosed with tuberculosis, and two patients (0.6%) were under surveillance follow-up. Only one patient (0.3%) was found to have a lung mass, which was later confirmed as primary lung adenocarcinoma after a CT-guided biopsy. Table 2 provides further details on these patient characteristics.

**Table 2:** Patients with lung mass/lung nodule >1 cm.

Cases	Case 1	Case 2	Case 3	Case 4	Case 5
Age	60 years	49 years	50 years	43 years	83 years
Gender	Female	Male	Male	Female	Female
Risk factor	No risk factors	Smoking “40 pack-years” and pancreatic cancer	Smoking “45 pack-years”	No risk factors	Second-hand smoke
Comorbidity	Diabetes mellitus type 2	Dyslipidemia, ischemic heart disease, ampulla of Vater adenocarcinoma in 2019, status post-Whipple procedure	Healthy	Diabetes mellitus type 2, hypertension, dyslipidemia	Second-hand smoke
Radiological characterization	Left supra hilar mass, 2.6 x 1.2 cm	Right lung base mass, 1.6 x 1.4 cm, and right lower lobe superior segment mass, 1.3 x 1.3 cm	Fibro-nodular infiltration in the upper lobe, 2.3 x 1 cm	Soft tissue density lesion seen in the left lower lobe, 2 x 1.5 cm	Sub-pleural mass, irregular margin, lateral segment, left lower lobe, 3.2 x 3.1 cm
Symptoms	Asymptomatic	Dry cough, weight loss	Productive cough, night sweats, weight loss	Asymptomatic	Dry cough
Investigation	CT-guided lung biopsy	CT-guided lung biopsy	Acid-fast bacilli smear and culture from sputum		
Final diagnosis	Lung parenchyma showing very focal granuloma formation, no malignancy identified	Metastatic adenocarcinoma in keeping with the patient’s history of ampulla of Vater adenocarcinoma	Tuberculosis	Pleural fibroma	Possible primary lung cancer
Management	Planned for surveillance chest CT in 3 months as per lung multidisciplinary meeting recommendation	Palliative chemotherapy (gemcitabine and paclitaxel)	Rifampicin 600 mg daily for 6 months, isoniazid 300 mg daily for 6 months, pyrazinamide 1500 mg daily for 2 months, ethambutol 1200 mg daily for 2 months	Follow-up chest CT scan after 3 months	The patient declined any further investigation

**Figure 1:**
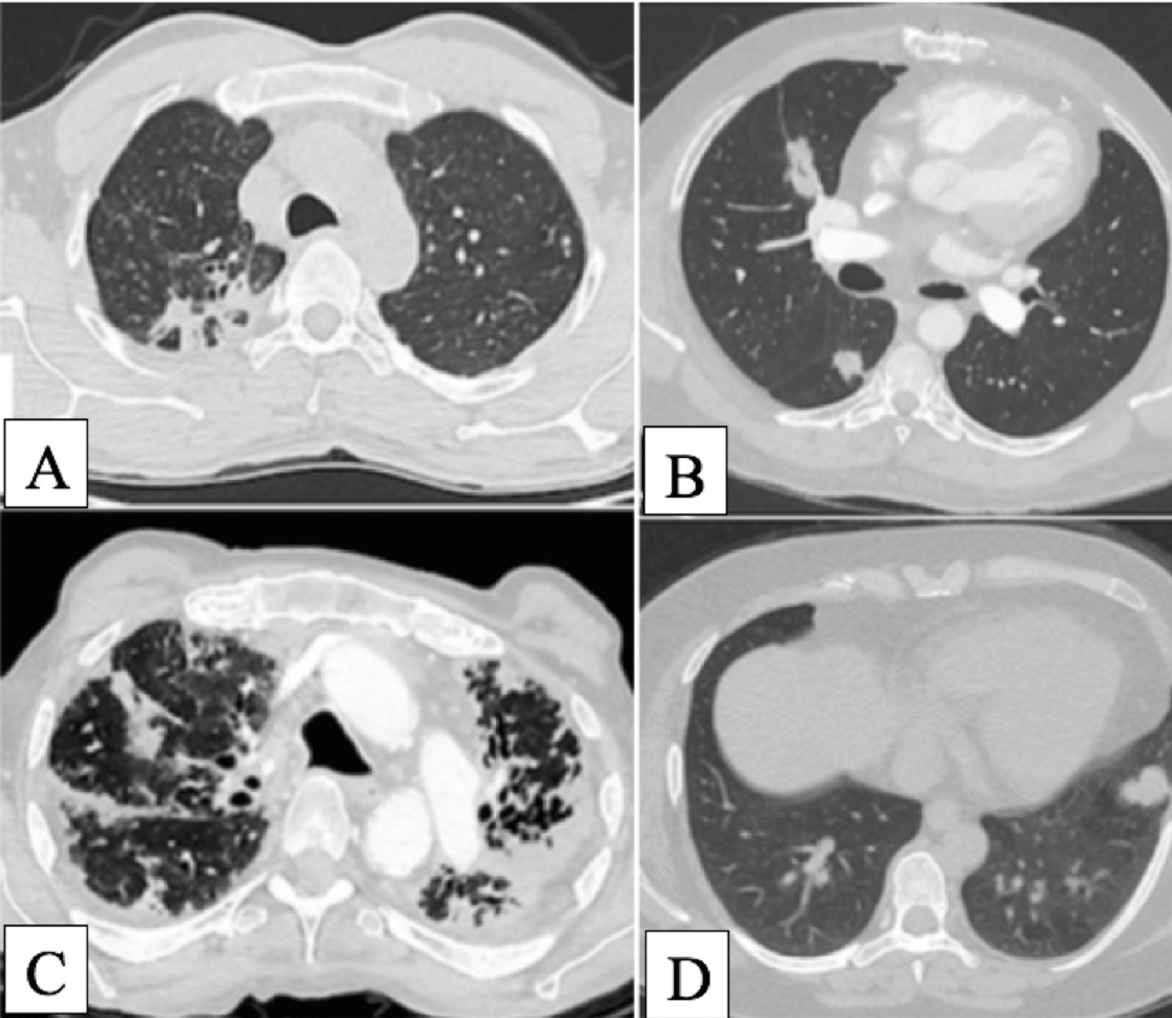
Incidental lung pathologies. A: Post-tuberculosis changes; B: lung nodule; C: chronic hypersensitivity pneumonitis; D: pleural fibroma.

Focusing on ILD, eight patients (2.5%) exhibited findings consistent with ILD. This included two cases of non-specific interstitial pneumonia (NSIP), five cases of usual interstitial pneumonia (UIP), and one case of hypersensitivity pneumonitis (HP) (Figure 1). All patients with ILD findings were evaluated for autoimmune-related ILD, but no autoimmune conditions were identified during follow-up. The characteristics of these patients are detailed in Table 3.

**Table 3:** ILD cases. ILD: interstitial lung disease; NSIP: non-specific interstitial pneumonia; PFT: pulmonary function test; 6-mWT: six-minute walk test; CRP: C-reactive protein; mg/dl: milligram per deciliter; ESR: erythrocyte sedimentation rate; ANA: antinuclear antibody; c-ANCA: antineutrophilic cytoplasmic antibodies; p-ANCA: perinuclear anti-neutrophil cytoplasmic antibodies; RF: rheumatoid factor; CCP: cyclic citrullinated peptide antibody; FeV1: forced expiratory volume in one second; IPF: idiopathic pulmonary fibrosis; UIP: usual interstitial pneumonia; FVC: forced vital capacity; DLCO: diffusing capacity of the lungs for carbon monoxide; TB: tuberculosis.

Cases	Age/Gender	Smoking status	ILD pattern	PFT	6-mWT	Inflammatory markers	Autoimmune antibodies: (ANA, RF, CCP, c-ANCA, p-ANCA)
Case 1	83/Female	2^nd^-hand smoker	ILD - Mixed cellular and fibrotic NSIP pattern	Not done	Not done	CRP: 45 mg/L	Negative
Case 2	76/Male	Ex-smoker	ILD - Post-inflammatory fibrotic changes due to previous infection	Mixed picture, predominant severe obstructive pattern with FeV1 = 47% (1.21 L)	Lowest O2 saturation = 96%, distance = 195 meters	CRP: 104 mg/L; ESR: 120 mm/hr	Negative
Case 3	71/Male	Ex-smoker	IPF-UIP pattern	Severe restrictive pattern with DLCO: 24%. FVC: 42% (3.3 L)	Lowest O2 saturation with exercise: 88%. Exercise distance: 200 meters	CRP: 26.8 mg/L; ESR:100 mm/hr	Negative
Case 4	73/Male	Ex-smoker	IPF-UIP pattern	Mild restrictive pattern with FVC: 75% (2.44 L)	Lowest O2 saturation with exercise: 84%. Exercise distance: 180 meters	CRP: 230 mg/L	Negative
Case 5	91/Male	Ex-smoker	IPF-UIP pattern	Severe restrictive pattern with DLCO: 27% and FVC: 46% (1.3L)	Not done	CRP: 30.2 mg/L	Negative
Case 6	78/Male	Falcon trainer	ILD - chronic hypersensitivity pneumonitis and post-TB bronchiectasis	Severe restrictive pattern with DLCO: 22% and FVC: 52% (1.84)	Not done	CRP: 99 mg/L; ESR: 105 mm/hr	Negative
Case 7	59/Male	Ex-smoker	ILD - post-inflammatory	Mixed picture, predominant mild obstructive pattern with DLCO: 52% and FeV1 = 85% (2.91 L)	Not done	CRP: 99 mg/L	Negative
Case 8	73/Male	Ex-smoker	ILD - early NSIP pattern	Normal spirometry	Not done	CRP: 30 mg/L	Negative

## Discussion

This cross-sectional chest CT-based study, which provides novel insights into incidental lung abnormalities, represents the first of its kind in the city of Al Ain, United Arab Emirates. The rapid increase in chest CT scans during the COVID-19 pandemic, largely driven by the need to assess pulmonary and other complications of the COVID-19 infection, created an unprecedented opportunity to observe incidental lung findings in a relatively large cohort. The prevalence of incidental chest findings (incidentalomas) in patients who underwent CT scans for suspected or confirmed COVID-19 was 10.6%. Notably, lung nodules, either single or multiple, comprised the most common incidental findings, accounting for 56% of all incidentalomas.

Our findings align with existing literature but highlight some discrepancies. Previous studies have reported a wide variation in the prevalence of chest incidentalomas, ranging from 22% to 46% in patients undergoing CT scans for various indications. These differences could be attributed to several factors. First, the timing of these studies during different waves of the COVID-19 pandemic likely influenced the nature and frequency of CT scans. For example, earlier waves of the pandemic may have included more patients with severe COVID-19 symptoms, who were more likely to undergo chest imaging, potentially leading to a higher detection rate of incidentalomas. Additionally, differences in patient demographics, clinical indications for imaging, and variations in scanning protocols across institutions may have contributed to the variability observed across studies [2-5].

Table 4 compares the findings of our study with recent studies conducted in other regions, highlighting both the similarities and the context-specific differences in the prevalence of incidental findings. Such comparisons are essential for understanding how local healthcare practices and population health factors might influence the prevalence and types of incidentalomas detected in different settings.

**Table 4:** Comparison of the present study with recent similar studies. HRCT: high-resolution computed tomography.

Author’s name and year	Place of study	Sample size	Parameters compared	Conclusions
Sandeep et al. (2023) [4]	India	1,000	To evaluate the non-COVID-19 lung pathologies and other system findings in HRCT chest done for COVID screening study.	Finding incidentalomas could result in many costly follow-up exams, increased radiation exposure for healthy patients, needless invasive procedures, and patient concerns. The radiologist should provide evidence-based recommendations for additional workup or follow-up when reporting an accidental finding as well as the likelihood of the finding’s significance [4].
Dundar et al. (2021) [5]	Turkey	1,540	Parenchymal and extra-parenchymal findings	It is important to define incidental findings other than pneumonia in patients who underwent chest CT scans during the pandemic. The CT scans of the patients with a suspicion of COVID-19 must be examined in detail [5].
Ramanan et al. (2021) [6]	Chennai, India	Multicentric study	Incidental chest computed tomography findings in asymptomatic COVID-19 patients	The COVID-19 pandemic will take some time to run its course and, even after if it begins ebbing away, sporadic cases may show up as asymptomatic incidental findings on CT scans conducted for other indications. These findings represent occult community infection and need to be addressed swiftly [6].
Frank and Quint (2012) [7]	Michigan, United States	-	Chest CT incidentalomas: thyroid lesions, enlarged mediastinal lymph nodes, and lung nodules	Incidental findings are frequently found on chest CT examinations. Although most of these findings are not clinically significant, some may represent incidentally discovered malignancies [7].

There are, however, several limitations to our study. Firstly, it is a retrospective analysis, which may introduce inherent biases related to patient selection and data collection. Secondly, as a single-center study, our findings may not be entirely generalizable to other regions or populations in the UAE or beyond. The relatively small sample size further limits the statistical power of our analysis and our ability to detect less common incidental findings. Moreover, the retrospective design precludes any longitudinal follow-up of the incidental findings; meaning, we cannot assess the clinical significance or progression of these abnormalities over time. This highlights the need for future studies with larger, more diverse populations and prospective designs to better understand the implications of incidental chest findings in COVID-19 patients.

Finally, while our study contributes valuable local data to the global discourse on incidentalomas in the context of COVID-19, it also underscores the importance of refining clinical guidelines regarding the management of these findings. As incidentalomas may or may not have clinical significance, further research is required to develop strategies for follow-up, especially in cases where patients may be at risk of developing malignancies or other significant health issues.

## Conclusions

This cross-sectional chest CT study highlights the occurrence of incidental lung abnormalities. Among the participants, 10.6% were found to have incidental lung nodules, none of which were associated with autoimmune conditions related to ILD. The study, conducted on a diverse group of COVID-19-positive patients representative of the general population in Al Ain city, concludes that routine mass screening for ILD or malignancy is not recommended unless clinically indicated. While the primary objective was to assess the prevalence of incidental findings during COVID-19 screenings, it raises important questions about whether targeted screening of high-risk individuals for lung cancer or ILD could facilitate early detection and reduce mortality rates. Current guidelines, such as those from the American College of Radiology (ACR) and the Fleischner Society, emphasize a risk-based approach to lung cancer screening, primarily focusing on individuals with significant risk factors such as smoking history or family predisposition. Further studies are needed to evaluate the potential benefits of early detection strategies for other populations at risk for lung abnormalities.

Current debates focus on whether routine screening for ILD should be incorporated into general clinical practice or reserved for high-risk populations, such as smokers or those with a family history of lung disease. While some suggest targeted screening for early detection, especially for high-risk patients, the risks of overexposure to radiation and the uncertain progression of ILDs warrant a cautious approach.
